# Particular Distribution of *Enterobacter cloacae* Strains Isolated from Urinary Tract Infection within Clonal Complexes 

**DOI:** 10.7508/ibj.2016.01.007

**Published:** 2016-01

**Authors:** Majid Akbari, Bita Bakhshi, Shahin Najar Peerayeh

**Affiliations:** Dept. of Bacteriology, Faculty of Medical Sciences, Tarbiat Modares University, Tehran, Iran

**Keywords:** *Enterobacter cloacae* complex, Urinary tract infection, hsp60

## Abstract

**Background:**

Based on biochemical properties*, Enterobacter cloacae* represents a large complex of at least 13 variant species, subspecies, and genotypes that progressively identified as the most species causing hospital-acquired infections. The aim of this study was to determine the relevance between phylogenetically related strains within the *E. cloacae *complex and the frequency of urinary tract infection caused by them.

**Methods:**

A 268-bp fragment was obtained from *hsp60 *gene for 50 clinical *E. cloacae *isolates from urine cultures of inpatients that admitted to six hospitals in Tehran, Iran during December 2012 to November 2013. The 107 nucleotide sequences were analyzed and the evolutionary distances of sequences were computed and neighbor-joining tree was calculated.

**Results:**

It showed that all of the genetic clusters have not an equal involvement in pathogenesis of urinary tract infections. Three superior clusters were found, together representing more than two third (80%) of the isolates (cluster VI with 25 members; clusters III and VIII with 9 and 6 members, respectively) and some genetic clusters were absent (IV, X, XII, and xiii), some of which are supposed to be associated with plants and no human infection has been reported.

**Conclusions:**

This study, for the first time, reports the unequal contribution of *E. cloacae *complex subspecies and clusters in *urinary tract infection*s in Iran and together with studies from other countries suggest that the subspecies of *E.hormaechei subsp. Oharae* is the most prevalent *E. cloacae* complex subspecies regardless of country under study.

## INTRODUCTION

*Enterobacter *spp. are Gram-negative and facultative anaerobic bacteria that are saprophytic in the environment, as they are found in soil and sewage. These bacteria are also parts of the commensal flora of the human gastrointestinal tract and can be considered as pathogens of plants, insects, and humans^[^^1^^]^.

Taxonomy of the genus *Enterobacter* has been repetitively updated^[^^2^^]^. Within a genetic complex, referred to as the “*Enterobacter cloacae *complex”, six genetically related and phenotypically similar species have been merged, i.e. *E. cloacae*, *E. asburiae*, *E. dissolvens*, *E. hormaechei*, *E. kobei*, and *E. nimipressuralis*. Most of them share a DNA relatedness with *E. cloacae *ranging from 61 to 67%^[^^1^^]^. In addition to these species, at least six genetic clusters are phylogenetically defined within the complex^[^^3^^]^. These clusters are routinely identified as ‘*E. cloacae’* using commercial biochemical kits such as API20E*.* However, the exact identification of the isolates within this taxon is difficult. Analysis of the *16SrRNA* gene is widely used for bacterial recognition, but it is poorly discriminative for closely related members of the *Enterobacteriaceae* family and more specifically, for members of the *Enterobacter* genus^[^^4^^]^. Other targets for molecular recognition of the isolates within the *Enterobacter *genus are *oriC*^[^^5^^]^, *gyrB*^[^^6^^]^, *rpoB*^[^^3^^,^^7^^]^, and *hsp60 *locus^[^^3^^]^. The *hsp60* gene sequencing-based identification seemed to be both discriminatory and easily carry out; however, for the identification of Enterobacter, other sequence-based molecular methods were not as accurate. Sequence analysis of *rpoB* and DNA *gyrB* genes were also supposed to be discriminatory for *E. cloacae* complex, but the lack of unanimity and consistency for the analysis of results has led us and other investigators to consider *hsp60* as a powerful tool for assigning the sub-species and clusters^[^^6^^]^.

Sequence analysis of a segment of the *hsp60 *gene demonstrated that the *E. cloacae *complex could be divided into 12 genetic clusters (I to XII) and one sequence crowd (*xiii*)^[^^4^^,^^8^^]^. Their specific names of some of these clusters are as follows: *E. asburiae *(cluster I), *E. kobei* (cluster II), *E. ludwigii* (cluster V), *E. nimipressuralis* (cluster X),* E. cloacae *subsp. *cloacae *(cluster XI), and *E. cloacae* subsp. *dissolvens *(cluster XII)^[^^3^^,^^8^^,^^9^^]^. However, the name *E. hormaechei* was sometimes applied as a generic name for strains belonging to various* hsp60* gene sequencing-based clusters, including VI, VII, and VIII, which are related to three subspecies: *oharae, hormaechei*, and *steigerwallti*, respectively^[^^10^^]^. Species names were not ascribed to clusters III, IV, and IX and to sequence crowd *xiii*.

Based on biochemical properties, what we identify in the laboratory as *E. cloacae* represents a large complex of at least 13 variant species, subspecies, and genotypes. *E. cloacae *has been progressively identified as the 10 most frequent species causing hospital-acquired wound, pneumonia, urinary tract infections, and sepsis in intensive care units. In spite of this relevancy, little is known about the correlation between phylogenetically related strains within the *E. cloacae *complex and the frequency of diseases caused by these^[^^11^^]^. The members of the *E. cloacae* complex differ in pathogenicity to humans, and some members have been reported to cause an epidemic outbreak^[^^12^^]^.

To our knowledge, there are no published data on molecular epidemiology of *E. cloacae* genotypes in Iran. Therefore, the current study was conducted to identify the specific distribution within the *E. cloacae* complex of strains isolated from the patients with urinary tract infection in Tehran, Iran.

## MATERIALS AND METHODS

**Bacterial strains and epidemiological data**

In total, 50* E. cloacae *isolates were collected from urine cultures of inpatients suffering from urinary tract infection admitted to six large academic and governmental hospitals in Tehran, Iran during December 2012 to November 2013. The samples were systematically and prospectively collected and stored. The identification of the infecting organism as *E. cloacae *was confirmed using a routine phenotypic identification system (the API20E, BioMerieux, France). *E. cloacae* PTCC (Persian Type Culture Collection) 1003 and *E. cloacae* PTCC 1798 were used as standard strains.

**Molecular identification methods**

Bacterial DNA was prepared for PCR analysis with using boiling method in which fresh bacteria colonies were suspended in 500 μl sterile distilled water (molecular grade) and boiled for 10 minutes. The suspension was centrifuged (at 10,000 ×g at room temperature for 10 min), and the 200-μl supernatant was transferred to a microtube and used directly for PCR assay. Partial sequencing of the *hsp60 *gene was performed by a previously described protocol^[^^3^^]^. Briefly, oligonucleotide primers Hsp60-F (5_-GTAGAAGAA GGCGTGGTTGC-3_) and Hsp60-R (5_ATGCATT CGGTGGTGATCATCAG-3_) were used for genomic amplification of a 341-bp fragment of the *hsp60 *gene. Amplification was also performed in a reaction mixture with total volume of 25 μl, containing 15.6 μl sterile water (molecular grade), 2.5 μl 10× Taq polymerase buffer, 0.3 μl dNTPs (10 mmol l^-1^), 0.5 U Taq DNA polymerase, 25 pmol each primer, 0.6 µL MgCl_2 _(50 mM), and 5 μl template DNA. Amplification was performed as follows: initial denaturation step at 94°C for 5 min, followed by 30 cycles consisting of denaturation (94°C for 30 s), annealing (57°C for 30 s) (separately adjusted for each set of primer pairs), and extension (72°C for 60 s) with a final extension step at 72°C for 5 min. PCR products were visualized on 1% agarose gels after electro-phoresis and staining. PCR was performed. Forward strand of the amplified DNA fragment was used for direct sequencing using the ABI 3730X capillary sequencer (Genfanavaran, Macrogen, Seoul, Korea).

**Nucleotide sequence accession numbers**

The sequences of the following reference and type strains were retrieved from the GenBank database. The number of genotype and reference strains of *E. cloacae *complex in Table 1 refer to Hoffmann and Roggenkamp study^[3]^. A 268-bp sequence of the *hsp60 *gene was obtained from 50 clinical and 2 PTCC strains. The sequences were compared with 44 reference sequences from the strains previously described in taxonomic studies by Hoffmann and Roggenkamp^[^^3^^]^, four reference sequences for every cluster except cluster X that has 1 sequence, and 11 sequence type strains^[^^3^^,^^13^^]^. All of the 50 clinical and 2 PTCC sequences were deposited in the GenBank under the accession numbers KM202107, KM222360 through KM222408, KM278223 and KM278224. 

**Table 1:** Reference and type strains retrieved from the GenBank database and used in this study

**Reference/ ** **type strains**	**Species **	**Nucleotide sequence accession numbers **
Cluster I	*E. asburiae*	(AJ567893.1, AJ567846.1, AJ417140.1, **AJ417141.1**)
Cluster II	*E. kobei*	(AJ567888.1, AJ567886.1, AJ567862.1,AJ567849.1)
Cluster III	*E. cloacae *III	(AJ567880.1, AJ567877.1, AJ567872.1, AJ567871.1)
Cluster IV	*E. cloacae* IV	(AJ543893.1, AJ543807.1, AJ543889.1, AJ543877.1)
Cluster V	*E. ludwigii*	(AJ862859.1, AJ862861.1, AJ862862.1, AJ862863.1)
Cluster VI	*E. hormaechei subsp. oharae*	(AJ567891.1, AJ567885.1, AJ567878.1, AJ567876.1)
Cluster VII	*E. hormaechei subsp. hormaechei*	(AJ866491.1, AJ862866.1, AJ862867.1,AJ417108.1)
Cluster VIII	*E. hormaechei subsp. steigerwaltii*	(AJ567892.1 , AJ567890.1, AJ567889.1,AJ567884.1)
Cluster IX	*E. cloacae* IX	(AJ543878.1, AJ543819.1, AJ543881.1, AJ543820.1)
Cluster X	*E. nimipressuralis*	(**AJ567900.1**)
Cluster XI	*E. cloacae subsp. cloacae*	(AJ543855.1, AJ417139.1, **AJ417142**.1, AJ543768.1)
Cluster XII	*E. cloacae subsp. dissolvens*	(**AJ417143.1**, AJ862872.1, AJ543817.1, AJ543847.1)
Cluster xiii	*E. cloacae *sequencecrowd	(AJ543872.1, AJ543870.1, AJ417128.1, AJ543837.1)
Outgroup	*E. cancerogenus*	(ATCC 33241, **AJ567895**)
Outgroup	*E. amnigenus*	(ATCC 3072, **AJ567894**)
Outgroup	*E. cowanii*	(ATCC 107300T, **AJ567896**)
Outgroup	*E. gergoviae*	(ATCC 33028, **AJ567897**)
Outgroup	*E. pyrinus*	(ATCC 49851, **AJ567901**)
Outgroup	*C. sakazaki*	(ATCC 29544, **AJ567902**)

Type strains are shown in bold.

**Statistical analysis**

The evolutionary history was inferred using the Neighbor-Joining method^[^^1^^]^. The analysis involved 105 nucleotide sequences. The evolutionary distances computed using the Maximum Composite Likelihood method^[^^3^^]^ are in units of the number of base substitutions per site. There were a total of 268 positions with 74 variables in the final dataset. Evolutionary analyses were conducted in MEGA (version 6)^[^^14^^]^.

## RESULTS

**Prevalence of species and genotypes**

**Fig. 1:**
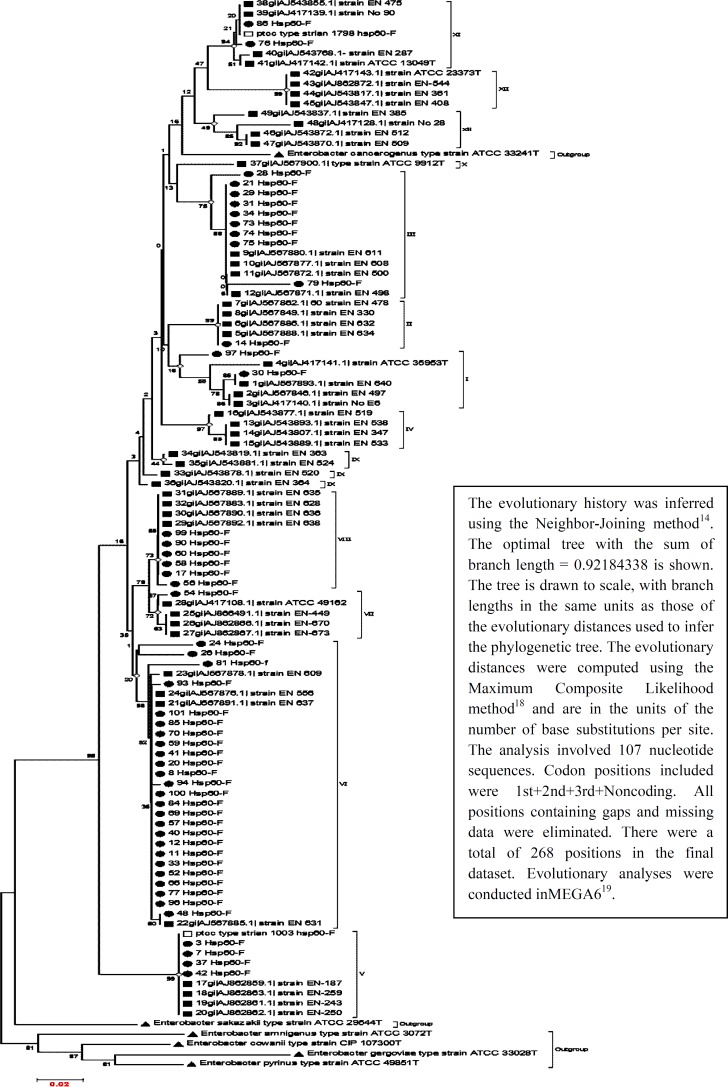
The evolutionary history of analysis 268 nucleotides (74 variable) of the *hsp60 *gene sequences from 50 clinical strains (urine) and 44 reference and 11 type strains of the genus *Enterobacter *(105 nucleotide sequences ) was inferred using the Neighbor-Joining method^[^^15^^]^. The optimal tree with the sum of branch length = 0.92184338 is shown. The tree is drawn to scale, with branch lengths in the same units as those of the evolutionary distances used to infer the phylogenetic tree

Each isolate was allotted to its individual species, subspecies, or genotypes by sequence analysis of 268-bp *hsp60*. A neighbor-joining tree was calculated, including all clinical, types, and reference strains of the *E. cloacae* complex as well as type strains of the *Enterobacter *genus (105 nucleotide sequences). The optimal tree with the sum of branch length = 0.92199563 is shown in Figure 1. The percentage of replicate trees in which the associated taxa clustered together in the bootstrap test (100 replicates) is shown next to the branches. The tree is drawn to scale, with branch lengths in the same units as those of the evolutionary distances used to infer the phylogenetic tree. Of 13 genotypes and species delineated so far, 8 were found in the present study. 

Thirty two (64%) isolates belonged to three *E. hormaechei* subspecies, explaining* E. hormaechei* by far the most eminent species of our study collection. Furthermore, only one isolate (2%) clustered with the *E. hormaechei* type strain (*E. hormaechei* subsp. *hormaechei*), 25 isolates (50%) were identified as *E. hormaechei* subsp. *Oharae*, and 6 isolates (12%) as *E. hormaechei* subsp. *Steigerwaltii*, suggesting that *E. hormaechei* subsp. *oharae* was the subspecies with the highest clinical relevance in *urinary tract infection.*

Nine isolates (18%) clustered within genotype III, making it the second most frequent genotype of the *E. cloacae* complex. In addition, 4 isolates (8%) grouped with cluster V (*E. ludwigii*). *E. cloacae *subsp. *cloacae*, *E. asburiae*, and *E. kobei *(clusters XI , I , II, and VII, respectively) were found in 2 (4%), 2 (4%), 1 (2%), and 1 (2%) respectively, while clusters IV, IX, X (*E. nimipressuralis*), XII (*E. cloacae *subsp. *dissolvens*), and xiii (*E.* *cloacae* sequence crowd) were absent among the isolates in this study (Table 2).

**Table 2:** Distribution of clinical strains within the genetic clusters of the *E. cloacae* complex

**Strains **	**Cluster**	**No. of** **urinary strains**	**Frequency (%)**
*E. asburiae*	I	2	4
*E. kobei*	II	1	2
*E. cloacae *III	III	9	18
*E. lodwigii*	V	4	8
*E. hormaechei subsp. oharae*	VI	25	50
*E. hormaechei subsp. hormaechei*	VII	1	2
*E. hormaechei subsp. steigerwaltii*	VIII	6	12
*E. cloacae subsp. cloacae*	XI	2	4
Total	8	50	100

## DISCUSSION

The current study demonstrates distribution of the strains involved in urinary tract infection within the genetic clusters of the *E. cloacae* complex. All of the 50 isolates, which were phenotypically identified as *E. cloacae* using API 20E, harbored the *hsp60* gene. It can be suggested that API 20E is a reliable tool for primary identification of *E. cloacae* complex; however, it should not be ignored that only sequence-based methods could discriminate the genotypes and clusters. 

On the basis of *hsp60* analysis, we showed that all of the genetic clusters have not equal involvement in the pathogenesis of urinary tract infections. Therefore, this fact underlines the necessity for more accurate, routine methods for bacterial identification and for better understanding of the etiology of *urinary tract infection* and pathogenesis of the *E. cloacae *complex^[^^11^^,^^13^^]^. Three superior clusters were found, together with representing more than two third (80%) of the isolates (cluster VI with 25 members and clusters III and VIII with 9 and 6 members, respectively). Other investigators from Euroupe have shown that the *E. hormaechei subsp. oharae* (cluster VI), * E. cloacae cluster *III, and *E*. *hormaechei* subsp. *steigerwaltii* (cluster VIII) are the species most frequently recovered from clinical specimens^[^^3^^,^^11^^,^^13^^]^. This finding suggests the equivalent distribution of genotypes in different geographical locations.

Morand and colleagues^[^^13^^]^ indicated that the most common genetic clusters were clusters III, VI, and VIII, which was detected among clinical strains routinely identified as *E. cloacae *in the clinical laboratory. However, clusters VI and VIII (*E.* *hormaechei*) but not cluster III had a dominant association with the infections of orthopedic implants and specifically, with the infected material in the hip (*P*=0.019)^[^^13^^]^.

Analysis based on microarray comparative genomic hybridization (CGH) showed two genetically distinct clades. Most strains related to the clinical diseases belonged to the youngest CGH-based clade (clade 2), which comprises clusters III, VI, and VII based on *hsp60 *gene sequencing. The second older clade (clade 1) includes heterogeneous strains, some of which are commensal^[^^12^^]^. Therefore, it can be inferred that Iranian *urinary tract infection*, which is prevalent in isolated strains, are derived from the younger CGH clade.

Paauw *et al.*^[^^12^^]^ reported *Enterobacter hormaechei *outbreak strain as the cause of a nationwide outbreak in the Netherlands, which carried a wide range of virulence and antimicrobial-associated genetic elements^[^^12^^]^. In total, 32 cases (64%) of *E. hormaechei* subspecies (clusters VI, VII, and VIII) in our study were dominant, indicating an outbreak of hospital-acquired *urinary tract infection* in Tehran that Pulsed-field Gel Electrophoresis genotyping findings rejected the hypothesis (unpublished data). Our data together with the finding of Paauw *et al.*^[^^12^^]^ emphasizes the need for routine monitoring of *E. cloacae* genotypes and clusters within the clinical isolates due to fear of clonal dissemination or outbreak occurrence. 

*Enterobacter asburiae *is a normal flora in the gastrointestinal tract that also is separated in water and soil. Also, it is most usually found in immune-compromised patients and associated with antibiotic use, enervated states, and chronic respiratory conditions^[^^15^^]^. Two strains of this organism isolated in our study were separated from two female newborns in two different hospitals, which further supports that *E. asburiae* is associated with immunocompromised patients newborns (in this study).

In the presrnt study, some genetic clusters IV, X, XII, and xiii were absent. These strains, which are mainly less prevalent, are found in nature. Cluster XII (*E. cloacae* subsp. *dissolvens*) is associated with plants and no human infections^[^^8^^,^^16^^]^. Cluster X (*E. nimipressuralis*) is found in potable water reservoirs but, to our knowledge, it has never been associated with human disease^[^^17^^]^.

In conclusion, this study, for the first time, reports the unequal contribution of *E. cloacae *complex subspecies and clusters in urinary tract infections. These results are consistent with other similar studies and suggest that the subspecies of *E. hormaechei subsp. Oharae* is the most prevalent *E. cloacae* complex genotype regardless of country under study. Other genotyping methods could be beneficial to assess the clonal correlation of strains within one *E. cloacae* cluster, which is mandatory for outbreak monitoring.
